# Spontaneous Resolution of Hemifacial Spasm Following Falcotentorial Meningioma Resection: Impact of Posterior Cranial Fossa Volume Changes on Hemifacial Spasm

**DOI:** 10.7759/cureus.85450

**Published:** 2025-06-06

**Authors:** Akihito Hashiguchi, Yushin Takemoto, Ryuta Ueda, Koichi Moroki, Hajime Tokuda

**Affiliations:** 1 Neurological Surgery, Tokuda Neurosurgical Hospital, Kanoya, JPN; 2 Neurosurgery, Kumamoto Red Cross Hospital, Kumamoto, JPN; 3 Neurological Surgery, Minami-Fukuoka Neurosurgical Hospital, Fukuoka, JPN

**Keywords:** hemifacial spasms, meningioma, posterior cranial fossa tumor, posterior cranial fossa volume, spontaneous resolution

## Abstract

A 56-year-old, previously healthy woman underwent imaging evaluation for nuchal pain, which revealed a falcotentorial meningioma measuring approximately 3.6 cm in maximum diameter. The tumor caused mild compression of the cerebellum and brainstem, and the nuchal pain was attributed to foramen magnum syndrome associated with the lesion. However, the patient had also experienced right hemifacial spasm (HFS) for several years. Initially, tumor resection alone was performed, and remarkably, the right HFS was found to have spontaneously resolved seven years later. Given that the spontaneous remission of HFS is exceedingly rare, we report this case along with a literature review addressing the clinical course and potential underlying mechanisms responsible for the natural resolution of HFS.

## Introduction

Hemifacial spasm (HFS) is a chronic neurological disorder characterized by uncontrollable contractions of the facial muscles on one side, typically caused by arterial compression at the facial nerve's root exit zone (REZ) [1]. In rare cases, secondary HFS caused by posterior cranial fossa (PCF) tumors has been reported, and such cases are often accompanied by significant displacement or twisting of the brainstem and cerebellum due to large tumors [2-7]. In the present case, despite the lack of direct intervention for HFS itself, the symptoms gradually improved over seven years following the surgical removal of a non-large falcotentorial meningioma that was discovered during the evaluation of nuchal pain. This case represents a rare example suggesting that changes in the volume of the PCF may have contributed to the resolution of HFS, thereby providing new insights into the onset of the disorder.

## Case presentation

**Figure 1:**
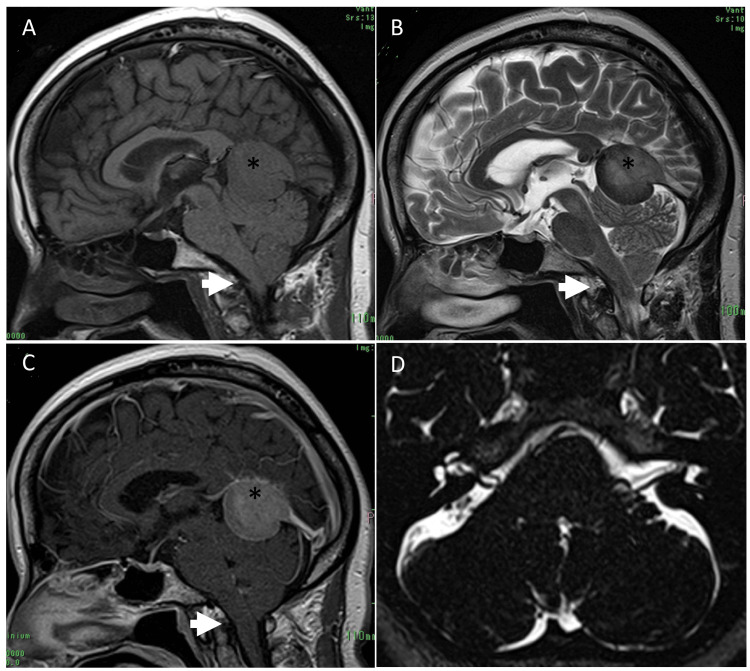
Preoperative magnetic resonance (MR) images (A, B, C) are all sagittal views, showing T1-weighted, T2-weighted, and contrast-enhanced T1-weighted images, respectively. A falcotentorial meningioma (asterisks) is lightly compressing the superior surface of the cerebellum, resulting in overcrowding at the foramen magnum (arrows). (D) is an axial view obtained with constructive interference in steady-state (CISS), demonstrating bilateral narrowing of the cerebellopontine angle cistern; however, no twisting or lateral displacement of the cerebellum or brainstem is evident.

A 56-year-old woman with no significant past medical history presented to our hospital with persistent nuchal pain as her chief complaint. By the time of evaluation, her nuchal pain had diminished; however, she reported a several-year history of right-sided HFS involving the orbicularis oculi, orbicularis oris, and platysma muscles. Magnetic resonance imaging (MRI) revealed a 3.6 cm long extramedullary mass in the anterior falcotentorial region. The tumor appeared hypointense on T1- and T2-weighted MRI with faint homogeneous enhancement and concomitant dural tail sign. The tumor caused slight downward displacement of the cerebellum and brainstem, resulting in mild overcrowding at the foramen magnum. However, no hydrocephalus, lateral shift, or twisting of the cerebellum and brainstem was observed. The cerebellopontine angle (CPA) cistern had narrowed. The anterior inferior cerebellar artery (AICA) was suspected of being the offending vessel responsible for the HFS (Figure 1).

**Figure 2:**
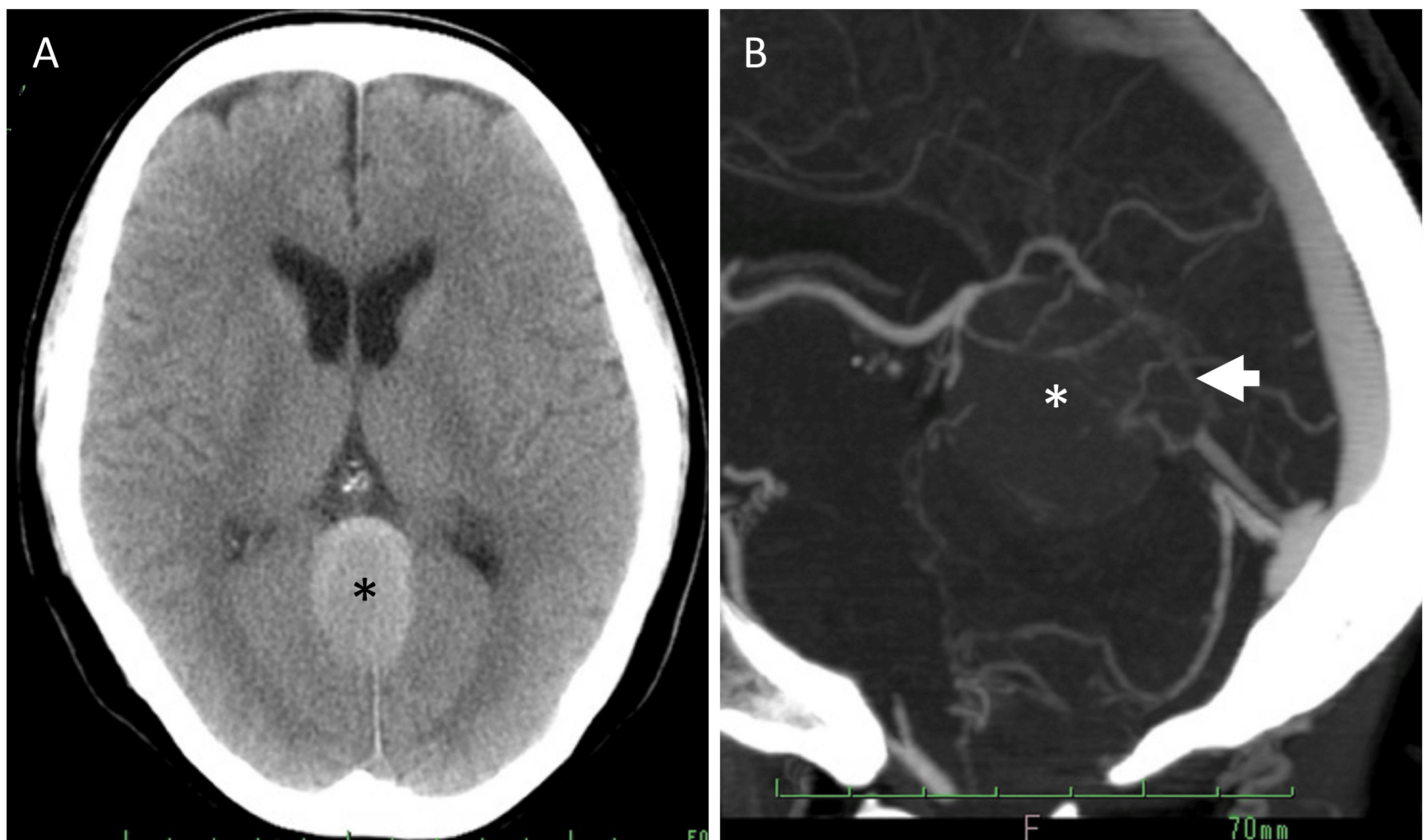
Preoperative computed tomography (CT) images (A) is a plain CT scan, and (B) is CT angiography. In (A), a falcotentorial meningioma (black asterisk) presenting as a hyperdense area is observed, although no hydrocephalus is seen. In (B), the tumor (white asterisk) shows minimal enhancement, and the straight sinus (arrow) is not visualized.

Computed tomography (CT) identified the tumor as faintly hyperdense. It was preoperatively diagnosed as a falcotentorial meningioma. CT angiography demonstrated no apparent tumor-feeding arteries and poor visualization of the straight sinus (Figure 2).

**Figure 3:**
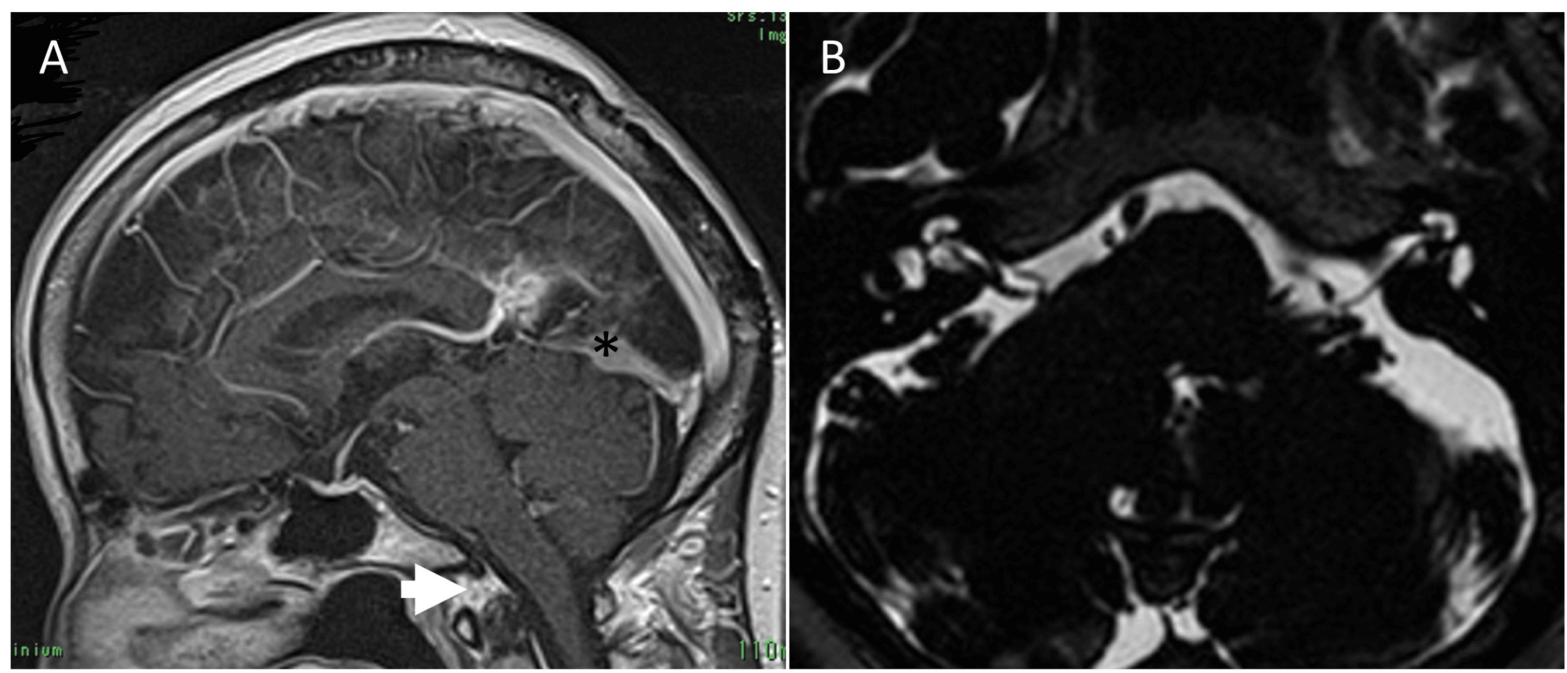
Postoperative magnetic resonance (MR) images Postoperative MR images were taken seven years after surgery. (A) is a sagittal contrast-enhanced T1-weighted image, and (B) is an axial image acquired using constructive interference in steady-state (CISS). The residual tumor (asterisk) did not increase in size after seven years postoperatively. The improved overcrowding at the foramen magnum (arrow) has also remained without recurrence. In the CISS image, the narrowing of the cerebellopontine angle cistern has not recurred.

The patient's nuchal pain was attributed to foramen magnum syndrome secondary to the tumor. Surgical resection of the tumor was prioritized as the initial intervention. Under general anesthesia, the patient was positioned prone, and gross total resection of the tumor was performed via a right-sided occipital interhemispheric approach (fibrous meningioma, World Health Organization grade 1). The postoperative course was uneventful. Postoperative contrast-enhanced MRI revealed a minimal residual tumor at the posterior tumor-straight sinus interface. However, the tumor's mass effect resolved, and the overcrowding of the foramen magnum improved substantially. The patient was discharged on postoperative day 10 and continued routine MRI follow-up. Despite the absence of specific interventions for her HFS, the symptoms gradually improved postoperatively and entirely resolved seven years after surgery. At the seven-year follow-up MRI, no tumor growth was observed, and the downward displacement of the brainstem and cerebellum or the crowdedness at the foramen magnum did not re-emerge. The narrowing of the CPA cistern had improved, but the preoperatively noted compression of the facial nerve's REZ by the right AICA appeared unchanged (Figure 3).

## Discussion

HFS is a common neuromuscular movement disorder characterized by brief or sustained involuntary contractions of muscles innervated by the facial nerve. Based on etiology, HFS is classified into primary and secondary forms. Primary HFS is diagnosed after excluding secondary causes such as tumors and demyelinating diseases; its primary etiology is considered to be neurovascular compression (NVC) at the facial nerve's REZ [1]. Primary HFS generally follows a progressive course, with spontaneous remission exceedingly rare. Although the initial symptoms are often mild, they worsen over time and significantly impact the patient's quality of life [8].

In contrast, only 0.4% of HFS cases are attributed to brain tumors [4]. Among CPA tumors, secondary HFS occurs in merely 0.44% (9/2050) of cases [5], underscoring the infrequent involvement of tumorous lesions. Various tumors, such as benign tumors arising in the CPA and brainstem gliomas, have been reported as rare causes of HFS. When tumors in the CPA are implicated, the proposed mechanisms include direct compression of the facial nerve by the tumor, compression in conjunction with adjacent vessels, circumferential encasement of the nerve by the tumor, direct compression by hypervascular tumors, or tumor-induced inflammation leading to thickening of the arachnoid membrane surrounding the nerve [5]. In cases of brainstem glioma, it is postulated that tumor enlargement results in compression of the facial nerve nucleus, thereby eliciting abnormal neural activity [9]. A particularly rare subset of cases involves tumors located remotely that induce HFS. To date, only seven such cases have been reported. These cases have involved relatively large tumors located at the contralateral CPA (five cases) [2,3,5], the foramen magnum (one case) [6], or the peritorcular tentorium (one case) [7], displacing the brainstem horizontally and causing narrowing of the CPA cistern. This displacement is thought to produce NVC, potentially leading to HFS. However, direct evidence of neurovascular contact on the affected side via imaging or intraoperative findings remains scarce, leaving some aspects of the pathophysiology unresolved. In our case, preoperative MRI demonstrated that a falcotentorial meningioma with a maximum diameter of 3.6 cm had its inferior half positioned within the PCF, resulting in compression of the superior aspect of the cerebellum. Although a mild caudal displacement of the cerebellum and brainstem was observed, horizontal displacement was not evident. Postoperative MRI confirmed improvement in the foramen magnum syndrome; however, correction of the cerebellum and brainstem's caudal displacement was limited.

The PCF is a confined space that houses the cerebellum, brainstem, and numerous nerves and vessels, which generally function without interference. However, these structures may become closely approximated when a lesion arises within this area, leading to mutual interference and potential functional impairment. Several reports have shown that patients with HFS have a significantly smaller PCF volume than non-HFS cases and that the cerebrospinal fluid volume, crowdedness, and shape of the PCF may influence NVC, the onset of HFS, and treatment outcome [10-14], suggesting that a constricted PCF might increase the likelihood of interference between the facial nerve and the compressing vessel. Nevertheless, given that secondary HFS due to CPA tumors is extremely rare (0.44%) [5], the contribution of PCF volume narrowness to the development of HFS, while plausible, is likely limited.

In our case, the slight postoperative expansion of the PCF gradually relieved the contact between the facial nerve and the responsible vessel, ultimately resulting in the spontaneous resolution of the symptoms. This observation represents a rare instance that appears to demonstrate the relationship between PCF volume and HFS, warranting further investigation and reporting.

## Conclusions

We report a case in which hemifacial spasm associated with a crowding of the PCF due to a falcotentorial meningioma spontaneously resolved following tumor resection. This case represents a valuable example that suggests the relationship between PCF and HFS.
